# Supplementary figures and images for: Protein sequence optimization with a pairwise decomposable penalty for buried unsatisfied hydrogen bonds
Source: PLoS Comput Biol. 2021 Mar 8;17(3):e1008061. doi: 10.1371/journal.pcbi.1008061 (PMC7971855; doi:10.1371/journal.pcbi.1008061)

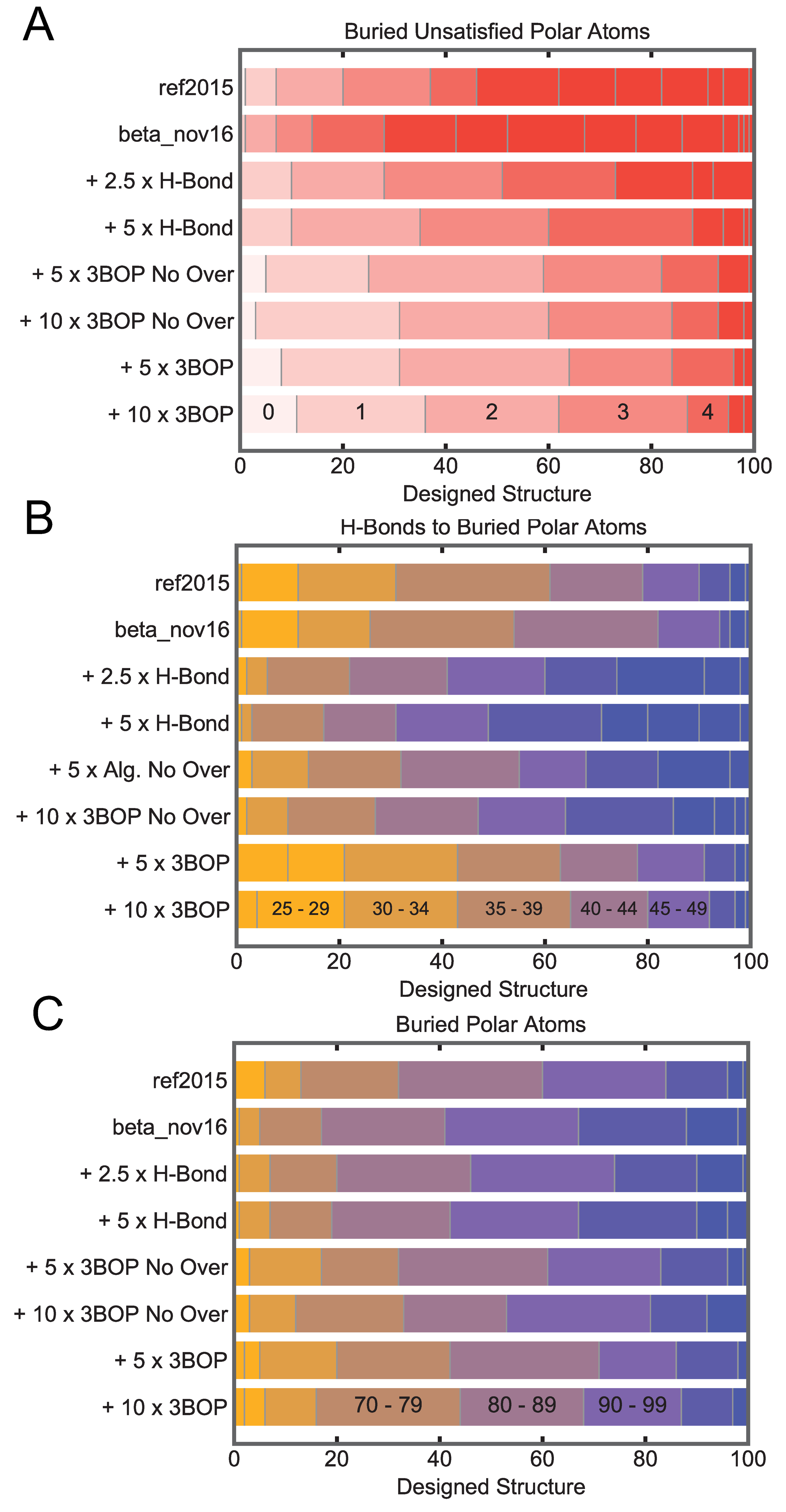

Supplement: S1 Fig — 100 native proteins were redesigned using the energy function and protocol described at the left of the panels, allowing only polar sidechains. Each method/row uses the same parameters as Fig 2A except Lysine-NZ used β = 15 and σ = -10 for the “5 x” variants and β = 30 and σ = -20 for the “10 x” variants. A) Number of buried unsatisfied polar atoms for each protein. In order from the left, vertical divisions indicate the number of proteins that have 0, 1, 2, or more unsatisfied polar atoms as indicated in the last row. B) Number of h-bonds to buried polar atoms. In order from left, each division represents the number of proteins that had from X to (X+4) h-bonds to buried polar atoms with each division to the right representing from (X+5) to (X+9). C) Number of buried polar atoms. In order from left, each division represents the number of proteins that had from X to (X+9) buried polar atoms with each division to the right representing from (X+10) to (X+19). For more information, see S1 Text. (TIF) [file pcbi.1008061.s001.tif]

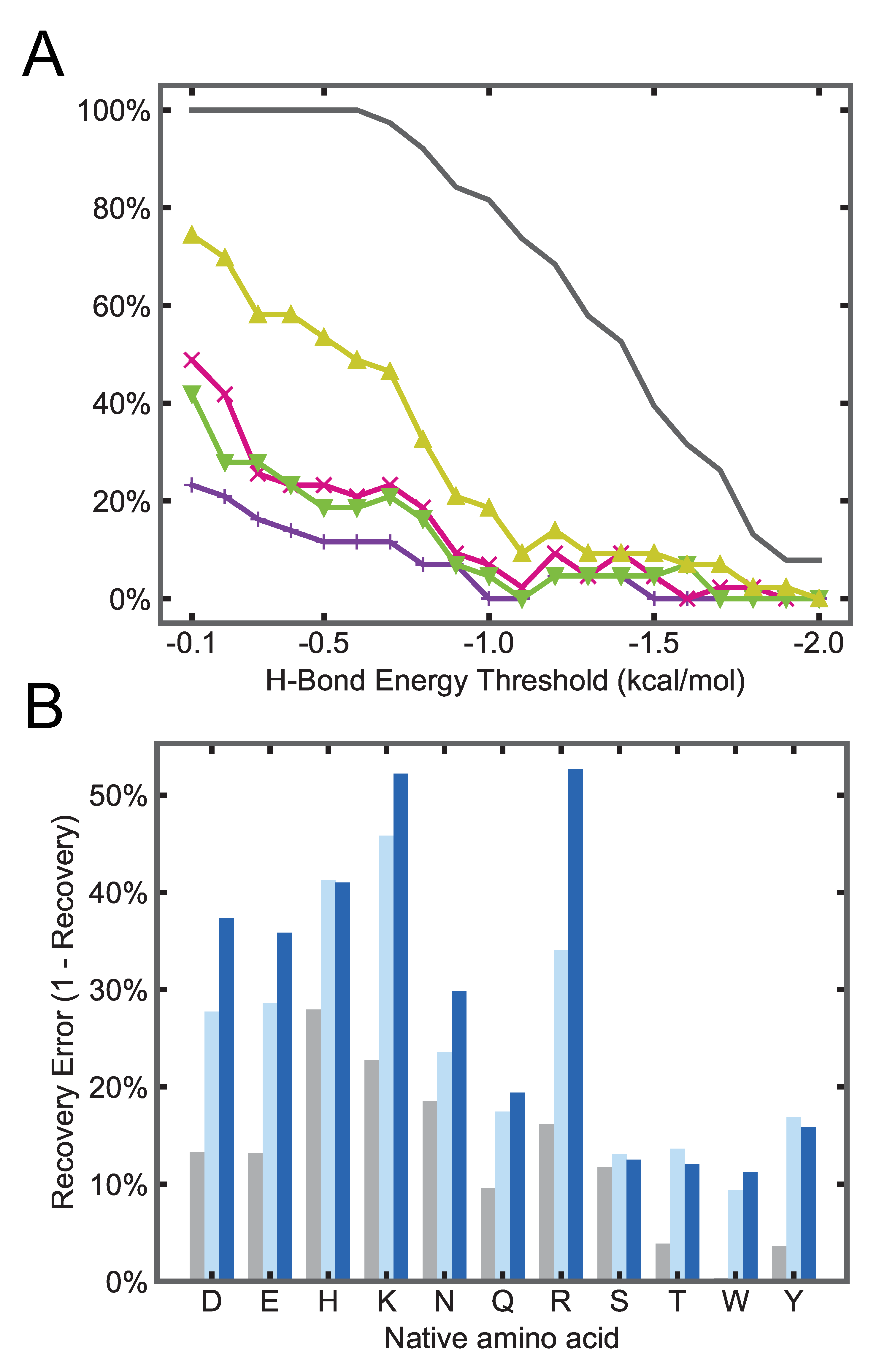

Supplement: S2 Fig — Extraneous oversaturation and performance A) The Outer Membrane Phospholipase A (PDB: 1ILZ) [15] was either repacked with standard rotamers (purple plus) or extra rotamers (pink cross) or redesigned with all amino acids using standard rotamers (green down arrow) or extra rotamers (yellow up arrow). An expansive buried h-bond network exists in the structure. The percentage of native rotamers in this h-bond network that experience extraneous oversaturation penalties to other native rotamers is plotted vs the energy threshold for a h-bond to be considered. In short, the extraneous oversaturation penalties were determined by performing the 3BOP algorithm and looking for penalties between native rotamers that were not present before the design/repack rotamers were considered (see S1 Text). The black line shows the percentage of h-bonds in the h-bond network that pass the energy threshold. B) Ninety-seven native proteins had their h-bond network residues redesigned using only polar amino acids. Amino acid recovery error of ref2015 (grey), ref2015 + 5 x 3BOP No Over (light blue), and ref2015 + 5 x 3BOP (dark blue) plotted. Parameters used for 3BOP tests identical to Fig 2 except Lysine-NZ used β = 15 and σ = -10. The h-bond threshold was set to -0.75. See S1 Text for details. (TIF) [file pcbi.1008061.s002.tif]

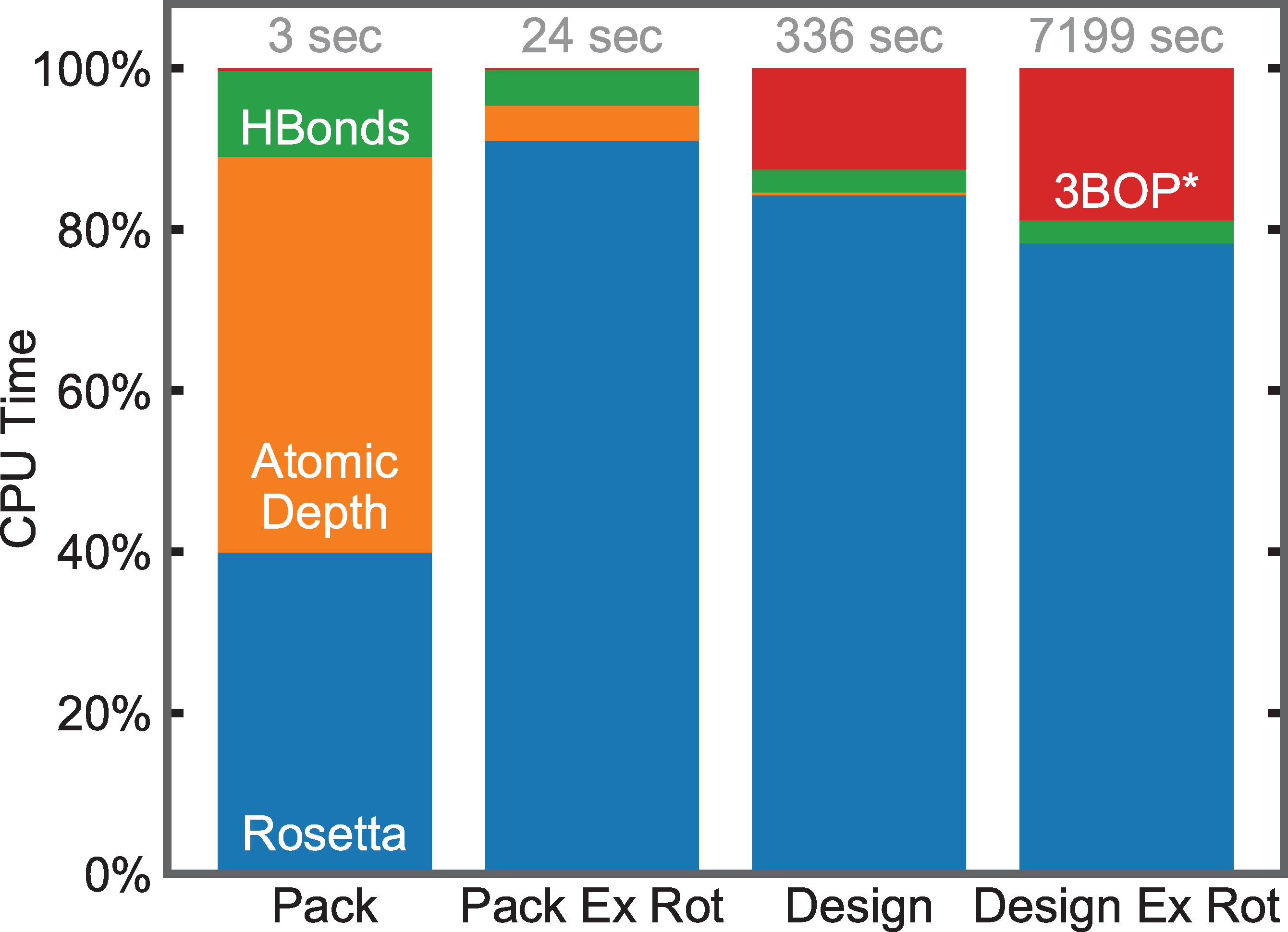

Supplement: S3 Fig — Each stacked bar graph represents the CPU time spent performing a packing or design calculation on 1ILZ using the pre-computed interaction graph setting. The red top bar represents time spent applying the penalty rules to rotamers, green bar represents time spent calculating h-bonds between rotamers before the 3BOP algorithm, the orange bar is time spent calculating atomic depth, and the blue bottom bar is runtime of the background packing or design calculation. With better data management, the green bar could be avoided as h-bonds are double calculated here (with the other calculation occuring inside blue). *While 3BOP adds a large runtime penalty here, only 2% of this runtime is spent calculating the actual 3-body interactions. 98% of the runtime is spent later in dictionary lookups during rotamer-pair energy assignment. (TIF) [file pcbi.1008061.s003.tif]
