# Supplementary material for: Protein sequence optimization with a pairwise decomposable penalty for buried unsatisfied hydrogen bonds
Source: PLoS Comput Biol. 2021 Mar 8;17(3):e1008061. doi: 10.1371/journal.pcbi.1008061 (PMC7971855; doi:10.1371/journal.pcbi.1008061)
Supplement: S1 Text — (PDF) [file pcbi.1008061.s004.pdf]

Native Recovery Results

To further investigate the effect of the extraneous oversaturation penalties, buried h-bond networks in ninety-seven native proteins were redesigned using ref2015 or ref2015 + 3BOP with and without the oversaturation penalty enabled ($\omega=0$ and $\omega\neq0$). S2 Fig B shows that for some amino acid types, the oversaturation penalty greatly increased the recovery error. The largest increase is for Arginine, with the next largest for Aspartate, Glutamate, Lysine, and Asparagine. The trend here suggests that the amino acids that make the most number of hydrogen bonds also incurred the most number of extraneous oversaturation penalties. The structures with designed with 3BOP, although not containing native amino acids, for the most part, still contained fully-satisfied networks.

Performance Results

S3 Fig shows the runtime-performance of an implementation of 3BOP as compared to the ref2015 energy function in Rosetta for packing. For very short packing trajectories, the atomic depth calculation dominates the runtime. However, since the atomic depth calculation takes a constant 1.5 seconds on this protein, once the number of rotamers grows, atomic depth is no longer a significant factor. Although the 3BOP algorithm is $O(n^3)$, this is not why it grows with runtime here as even in the longest test, the 3-body calculations only accounted for 2% of the extra runtime (data not shown). Instead, the actual all-by-all assignment of pair energies at a later step in packing accounts for 98% of the runtime due to dictionary lookups for all pairs. While this implementation was designed with the pre-computed interaction graph of Rosetta in mind, Rosetta has since switched to an on-the-fly interaction graph for large problems. The

3BOP implementation can be easily adapted to this (while still requiring the large up-front 3-body calculation), and since the vast majority of the runtime here came from rotamer-pair assignment, 3BOP's runtime will shrink proportional to the decrease in pair-considerations.

Burial representation for Fig 1B

The burial region from Fig 1B may easily be produced using PyRosetta[15].

```
import pyrosetta
from pyrosetta.rosetta.core.scoring.atomic_depth import AtomicDepth
pyrosetta.init()
pose = pyrosetta.pose_from_file( "1UBQ.pdb" )
depth = 4.5
probe_radius = 2.3
atomic_depth = AtomicDepth( pose, probe_radius, True )
atomic_depth.visualize_at_depth( depth, "burial.pdb" )
```

Data collection for S1 Fig A

PDB selection

For this test, it was desired to obtain 100 native PDB structures that were globular, densely packed, and between 90 and 100 amino acids. The globular and densely packed nature was desired in order to provide a large burial region and to exclude the possibility of water-filled cavities. The protein lengths were chosen to be as large as possible without requiring excessive computation time.

First, the PISCES[16] dataset was searched for proteins between 90 and 100 amino acids. Then, the atomic depth calculations were performed. Proteins that had voids at depths deeper than 3Å were removed. (This secondary depth was calculated with a 6Å probe.) Next,

the deepest atom in the protein was identified and proteins without an atom deeper than 7Å were removed.

Finally, all pairwise TMalign scores[17] were calculated and the results clustered using k-means to 100 clusters. The cluster centers were chosen for this test. The median cluster distance was 0.30 and the minimum distance was 0.62.

Here are the 100 pdbs and chain IDs chosen for this test:

```
1C5E_B, 1CY5_A, 1LLI_B, 1LXN_A, 1MB1_A, 1MN8_A, 1NQJ_A, 1ODV_A,
1TS9_A, 1U9P_A, 1V98_A, 1W3E_X, 1X1W_D, 1XE1_A, 2A66_A, 2C3W_A,
2CO5_A, 2CWY_A, 2DYJ_A, 2EBB_A, 2IL4_A, 2J9U_A, 2OQK_A, 2PKO_A,
2PR0_A, 2PTV_A, 2QAI_B, 2QRR_A, 2V1Y_A, 2V33_A, 2V76_A, 3DSG_A,
3FN1_A, 3FPN_B, 3G73_A, 3GUF_A, 3H7H_B, 3HES_B, 3HIX_A, 3HRL_A,
3IPJ_A, 3JRZ_A, 3JTO_A, 3K0X_A, 3KAV_B, 3KE2_B, 3KLW_B, 3LKL_A,
3LMO_A, 3MQI_B, 3MYZ_A, 3NWX_B, 3PD7_A, 3PQK_A, 3Q7T_B, 3QBY_A,
3T04_A, 3T9Z_B, 3UV0_A, 3ZFI_A, 4AMH_B, 4AVR_B, 4DP4_X, 4DRH_B,
4EBG_A, 4ES1_A, 4G4K_A, 4I68_A, 4I6S_B, 4JEG_B, 4JGU_A, 4KV2_A,
4MHV_A, 4MMG_A, 4MU7_A, 4N1L_A, 4NDT_A, 4OJG_A, 4PUG_A, 4U3Q_A,
4UD7_C, 4XQM_A, 4YY2_A, 4ZHP_A, 5B8D_A, 5DCM_B, 5DOM_A, 5ELU_A,
5FWZ_A, 5HDN_B, 5JMB_A, 5KLF_A, 5KNH_I, 5KUJ_A, 5TVI_V, 5U0J_B,
5YDF_A, 5YM6_B, 5YPP_B, 6G6S_A
```

Polar design calculations

The goal of this calculation was to show that 3BOP does indeed lead to fewer buried unsatisfied when compared to alternatives in a design situation where only polar residues were allowed. Great care was taken; however, in order to provide an unbiased test such that 3BOP was not artificially given an advantage. To this end, the test was performed with a very basic design script that generally follows the best principles for protein design with Rosetta. Additionally, careful selection of amino acid substitutions eliminated ways in which 3BOP often "cheats".

The design script consists of a single PackRotamers step with extra rotamers and a BuriedUnsatisfiedPolars filter and is named approx_polar_design.xml. The

BuriedUnsatisfiedPolars filter was obtained directly from the Best Practices from the RosettaCommons wiki[18] in order to prevent biases. For this calculation, the premise of “all polar heavy atoms need at least 1 h-bond” was used in setting the weights for the approximate_buried_unsat_penalty and for filtering with the BuriedUnsatisifedPolars filter.

The amino acid substitutions for this calculation were chosen very carefully. The goal was to only allow polar amino acids at each position without causing other issues. The two main issues to avoid were: 1) SER can self-satisfy to a helix backbone and 2) not all sequence positions can accept all polar residues. The issue that 1) creates is that SER provides a null-solution for helical backbones which 3BOP would likely exploit. This issue was mitigated with regards to 2) by only allowing SER at positions where ASN wouldn’t fit. The issue that 2) creates is that native GLY and ALA positions may need to be conserved because the next smallest polar atom, SER, does not fit. With all of these considerations in mind, the substitutions in S1 Table were allowed.

S1 Table. Substitutions allowed for polar design calculations

Native AA	Allowed AA
A	AS + HNQDERK
G	GS + HNQDERK
CIPSTV	S + HNQDERK
DEFHKLMNQRWY	HNQDERK

The following command was then used to design and score each of the 100 pdbs.

```
$ROSETTA3/bin/rosetta_scripts
      -parser:protocol approx_polar_design.xml
      -script_vars ex=1 hbond=$HBOND approx=$APPROX oversat=$OVR
sfxn=$SFXN
      -dalphaball $ROSETTA3/external/DAlpahBall/DAlphaBall.gcc
      -s <name of pdb>
```

Then, S2 Table describes the specific settings used for each protocol:

S2 Table: Settings for polar design calculations

Protocol	HBOND	APPROX	OVR	SFXN
ref2015	1	0	1	ref2015
beta_nov16	1	0	1	beta_nov16
+ 2.5 x H-Bond	3.5	0	1	ref2015
+ 5 x H-Bond	6	0	1	ref2015
+ 5 x 3BOP No Over	1	5	0	ref2015
+ 10 x 3BOP No Over	1	10	0	ref2015
+ 5 x 3BOP	1	5	1	ref2015
+ 10 x 3BOP	1	10	1	ref2015

After the design calculations, the BuriedUnsatisfiedPolars calculator was used to determine the number of buried unsatisfied polar atoms. With the settings chosen, the calculation proceeded as follows. All h-bonds with scores less than 0 were considered satisfying. A heavy atom was considered buried if it and its attached hydrogens had less than 0.01 $Å^2$ using a 1.1Å probe calculated analytically. Residues with more than 20$Å^2$ total sasa were excluded from the calculation to prevent buried unsats from odd surface rotamers. Finally, a buried unsatisfied polar atom is then an non-hydrogen atom that is considered buried, makes 0 hydrogen bonds, and is not excluded by the 20$Å^2$ total sasa rule. The total number of such atoms was then summed for each structure. This procedure was repeated 5 times for each structure and the median taken.

Data collection for S1 Fig B and S1 Fig C

The data for S1 Fig B and S1 Fig C was produced by running the script count_buried_hbonds.py on the outputs from S1 Fig A. For buried h-bonds, residues with more than 20Å^2 total sasa were excluded. Then all h-bonds reported by rosetta regardless of energy were counted. The median of the 5 outputs from S1 Fig A was reported. For buried polar atoms, any heavy-atom that could donate or accept an h-bond and that had less than 0.05Å^2 sasa was counted.

Data Collection for Fig 2A

This test sought to test the methods in a more constrained system where simply placing fewer polar atoms wouldn't lead to success. A one-sided interface design problem was chosen where "scaffold" proteins of variable sequence were docked to a protein of fixed sequence, such that the algorithms would need to design polar sidechains to satisfy the fixed sequence protein. For this test, barnase (PDB: 1BRS) was chosen as the native protein as this is a classic example of a polar interface. For the scaffold proteins, the first 10 proteins of each topology in rd4 were chosen from Rocklen et. al[9]. This resulted in 40 scaffold proteins coming from the 4 topologies tested in that paper: HHH, HEEH, EHEE, EEHEE.

Docking

The 40 scaffold proteins were first mutated to poly-valine using polyV.xml and the following command:

```
$ROSETTA3/bin/rosetta_scripts
```

```
-s *.pdb
-parser:protocol polyV.xml
```

Next, Patchdock[11,12] was used to dock the poly-valine scaffold proteins to the barnase interface. The following residues on barnase were selected as the “receptorActiveSite”:

```
27 B
56 B
73 B
83 B
87 B
102 B
103 B
```

And the following parameters were used for each dock. (The notable parameter here is that the output is clustered at 3.00Å RMSD):

```
receptorPdb barnase_relaxed.pdb
ligandPdb scaffold.pdb
protLib $PatchDock/chem.lib
log-file scaffold.log
log-level 0
receptorSeg 10.0 20.0 1.5 1 0 1 0
ligandSeg 10.0 20.0 1.5 1 0 1 0
scoreParams 0.3 -5.0 0.5 0.0 0.0 1500 -8 -4 0 1 0
desolvationParams 500.0 1.0
clusterParams 0.1 4 2.0 3.00
baseParams 4.0 13.0 2
matchingParams 1.5 1.5 0.4 0.5 0.9
matchAlgorithm 1
receptorGrid 0.5 6.0 6.0
ligandGrid 0.5 6.0 6.0
receptorMs 10.0 1.8
ligandMs 10.0 1.8
receptorActiveSite ActiveSite.list
```

After the docking was finished, the top 10 docks as ranked by Patchdock were chosen for design.

Interface Design

The goal of the interface design xml was to provide a realistic interface design scenario without creating any biases that would specifically help 3BOP. interface_design.xml was used to design the interfaces and features these aspects:

- Target backbone fixed, binder backbone flexible, rigid-body jump flexible, 0.5Å deviation CA coordinate constraints
- All sidechains further than 10Å from other chain fixed
- Target sidechains allowed to repack, binder sidechains allowed to mutate to all 20 amino acids
- Interface energy-edges upweighted 3x during packing
- Buried unsatisfied polar atoms measured by subtracting monomeric state from complex

The rationale behind upweighting the interface edges is that in this limited test set, it was desired that Rosetta would totally “fill-in” the interface rather than leave massive holes as it tends to do. In this way, Rosetta couldn’t take the easy way out and place poly-glycine. Additionally, it was chosen to allow Lysine-NZ to only require 1-2 h-bonds inside of 3BOP for this test. The idea was that the BuriedUnsatisfiedPolars filter only required Lysine-NZ to make one h-bond, so 3BOP was set to optimize to the same goal.

All four hundred interfaces were designed with interface_design.xml using the following command and the settings from S2 Table:

```
$ROSETTA3/bin/rosetta_scripts
      -parser:protocol interface_design.xml
      -script_vars ex=1 hbond=$HBOND approx=$APPROX oversat=$OVR
sfxn=$SFXN
      -dalphaball $ROSETTA3/external/DAlpahBall/DAlphaBall.gcc
      -s <name of pdb>
```

This execution logged the number of buried unsatisfied polar atoms in the interface which was used for Fig 2A.

Data Collection for Fig 2B

The InterfaceAnalyzer mover was included in interface_design.xml to count cross-interface h-bonds.

Data collection for S2 Fig A

To collect the data for this figure, the Rosetta implementation of 3BOP was run on a Rosetta-relaxed pdb structure of 1ILZ[14]. The algorithm dumped all of the calculations it performed and this data was subsequently analyzed by a python script. The total oversaturation penalty between the residues of the central hbnet was compared before and during packing in order to assess the effect of the other rotamers.

The initial relaxation was performed with this command:

```
$ROSETTA3/bin/relax
      -relax:constrain_relax_to_start_coords
      -relax:coord_constrain_sidechains
      -use_input_sc
      -flip_HNQ
      -no_optH false
      -score:weights ref2015_cart
      -set_weights coordinate_constraint 1.0
      -crystal_refine
      -s <name of pdb>
```

For this target, the probe radius for burial was increased to 4Å to account for the water cavity in the center (i.e. to consider it buried).

The central hbnet was identified by looking for residues whose last atom was buried to at least 4Å depth and made at least 1 hbond with energy -0.5 kcal/mol to another residue. The chosen residues of the central hbnet are as follows: 17, 35, 38, 39, 43, 45, 66, 68, 72, 91, 93, 97, 99, 102, 110, 111, 113, 115, 117, 135, 137, 141, 143, 145, 155, 157, 159, 161, 172, 174, 198, 213, 228, 236, 238, 240, 242, 247, 251, 256, 258, 266, 267

Next the following command was used to run the approx_pack.xml with all combinations of these substitutions:

```
THRESHOLD=[-0.1,-0.2,-0.3,-0.4,-0.5,-0.6,-0.7,-0.8,-0.9,-1.0,-1.1,-1.
2,-1.3,-1.4,-1.5,-1.6,-1.7,-1.8,-1.9,-2.0]
EX=[0,1]
RESTRICT=["",",restrict_to_repacking"]
$ROSETTA3/bin/rosetta_scripts
     -s 1ilz_0001.pdb
     -hbond_disable_bbsc_exclusion_rule
     -parser:protocol approx_pack.xml
     -script_vars threshold=$THRESHOLD ex=$EX restrict=$RESTRICT
     -out:levels
     core.pack.guidance_scoreterms.approximate_buried_unsat_penalty:
     500
```

The log from this run was captured and was analyzed with analyze_oversats.py. In summary, this script looks for all oversaturation penalties applied between the native rotamers of the hbnet. Before the packing trajectory, the pose is scored which is the baseline for comparison. Then the script determines the penalty between each native rotamer in the hbnet, and any rotamer that has an extraneous oversat is flagged for the graph.

Data collection for S2 Fig B

The test for S2 Fig B was designed to provide a near-worst-case scenario for the oversaturation penalty. Ninety-seven native proteins with buried h-bond networks were identified for this test and are listed, along with their h-bond network residues in s2figb_hbnet_res.dat (numbers correspond to sequence positions starting from 1). As the goal here was to identify a difference with the oversaturation penalty enabled, all residues besides the h-bond network residues were held fixed to their original amino acid identities, but allowed to repack. In this way, 3BOP would be less likely to find new h-bond networks involving those residues. When the h-bond network residues were allowed to change identities, the same rules from S1 Table were allowed in order to prevent the null-solution of poly-serine.

Each of the native proteins was relaxed 10 times with the relax command from S2 Fig A in order to provide slightly different starting backbones. The following command was then used to redesign the h-bond network residues using the parameters from S2 Table.

```
$ROSETTA3/bin/rosetta_scripts
      -parser:protocol hbond_network_redesign.xml
      -script_vars ex=1 hbond=$HBOND approx=$APPROX oversat=$OVR
sfxn=$SFXN
      -s <name of pdb>
```

Data collection for S3 Fig

Data collection for S3 Fig is very similar to that for S2 Fig A. The relaxed 1ILZ model was packed with approx_pack.xml using all 4 combinations of EX and RESTRICT above. THRESHOLD was set to -0.75. The total runtime of the trajectory was compared to another trajectory where everything was the same, except the approximate_buried_unsat_penalty was

disabled (control_pack.xml). Additionally, a flag was passed to output timing information of the atomic_depth, hbond calculation, and 3-body oversaturation penalty calculation portions. As the full discrepancy in runtime between the control and the test trajectories could not be explained by this additional timing information alone, any extra time was attributed to the algorithm (as it slowed down other parts of Rosetta).

This is the command that was used to generate the timing information with the EX and RESTRICT substitutions mentioned above:

```
$ROSETTA3/bin/rosetta_scripts
      -s 1ilz_0001.pdb
      -parser:protocol approx_pack.xml
      -script_vars threshold=0.75 ex=$EX restrict=$RESTRICT
      -out:levels
      core.pack.guidance_scoreterms.approximate_buried_unsat_penalty:
      400
      -precompute_ig
```

The control commands are the same as above but replacing the xml with control_pack.xml.

All tests were performed on an idle node with an AMD Epyc 7702P 64 core processor using Rosetta in single-core mode. Tests performed for direct comparison were run simultaneously and with 3 replicates.
