# Supplementary material for: Protein sequence optimization with a pairwise decomposable penalty for buried unsatisfied hydrogen bonds
Source: PLoS Comput Biol. 2021 Mar 8;17(3):e1008061. doi: 10.1371/journal.pcbi.1008061 (PMC7971855; doi:10.1371/journal.pcbi.1008061)
Supplement: Attachment — Submitted filename: Buried Unsat Letter to Reviewer.pdf [file pcbi.1008061.s007.pdf]

The authors thank the reviewer for the very detailed comments and suggestions. We feel that we have addressed all of the concerns listed and have a stronger paper because of it. Here are the responses in the same order as the original review.

1. We liked the name 3BOP and have added it throughout the paper to provide a meaningful way to talk about the algorithm.
2. We documented which parameters were used for all tests in the main-text and noted that our suggested setting is the 5 x variant. The reviewer was correct in that 3BOP achieved most of its reduction in buried polars by simply burying fewer polars. While this does make a point about the default behavior of ref2015 to bury polars without repercussion, the interface design test seemed like a stronger point. We finished the interface design test and this has replaced Fig 2 in the text. The original monomer design test remains but has been moved to the supplement.
3. The authors greatly thank the reviewer for this comment. Upon closer inspection, the OnTheFlyInteraction graph makes a few assumptions that conflicted with the assumptions made by the 3BOP Rosetta implementation. The changes needed to make 3BOP work with the OnTheFlyInteraction graph are modest and will be implemented soon. (Keeping track of rotamer pointers instead of rotamer indices.)
4. The original Fig 3A (now S3 Fig) was actually the first time the authors investigated the extent of the phantom atoms and thus shed much insight to us; however, the reviewer makes a good point that this graph only demonstrates that an issue exists and not what results because of it. In examining the rotamer pair distributions like the reviewer suggested, it became clear that identifying subtle changes in these distributions would be very difficult, and instead it was recognized that most of the effects were simply due to a “fear” of the algorithm to place Arginine. The text details the results, but in short, the

side chains with more polar atoms experienced more phantom atoms which is what would be expected.

5. The algorithm was fixed and we switched to β, σ, and ω like the reviewer suggested. Hopefully now the algorithm can be understood by all.

And again, thank you for the detailed review. All points were able to be addressed which should help the paper stand up against the test of time.
